# Supplementary material for: Heterogeneous Impact of Water Warming on Exotic and Native Submerged and Emergent Plants in Outdoor Mesocosms
Source: Plants (Basel). 2021 Jun 29;10(7):1324. doi: 10.3390/plants10071324 (PMC8309020; doi:10.3390/plants10071324)
Supplement: Supplementary file 1 [file plants-10-01324-s001.zip › plants-1247584-supplementary.pdf]

Plants

Online Supporting Information

for the manuscript:

Heterogenous impact of a water warming on exotic and native submerged and emergent plants in outdoor mesocosms

Morgane B. Gillard[1], Jean-Pierre Caudal[1], Carole Deleu[2], Gabrielle Thiébaut[1]

1 ECOBIO, UMR 6553 CNRS, Université de Rennes 1, Rennes, France

2 IGEPP, UMR 1349 INRA, Université de Rennes 1, Le Rheu, France

Plants

Supplementary Material 1 – Credits for plant silhouettes in Fig. 1-5

Ludwigia hexapetala silhouette – inspired by illustration by Marta Chirino Argenta in Flora acuática española. Hidrófitos vasculares (2014), with the author's authorization.

Myriophyllum brasiliense silhouette – derived from illustration by Massimiliano Lipperi in Identification guide of Invasive Alien Species of Union concern (2018), under free license.

Egeria densa silhouette - derived from illustration by Laura Line Reep for IFAS, Center for Aquatic Plants, University of Florida, with the institution's authorization.

Mentha aquatica leaves silhouette – inspired by illustration by Nastasic, under royalty-free license.

Myosotis scorpioides silhouette – inspired by illustration by Mark Mohlenbrock available in USDA-NRCS PLANTS Database / USDA NRCS, Wetland flora: Field office illustrated guide to plant species, USDA Natural Resources Conservation Service, under free license.

Potamogeton crispus silhouette – inspired by illustration by Dieter Tracey available in IAN/UMCES Symbol and Image Libraries, courtesy of the Integration and Application Network, University of Maryland Center for Environmental Science, under free license.

Supplementary Material 2 – Complimentary results and results of statistical analysis

Table S1. ANOVA results for individual final dry biomass in six species exposed to two temperature treatments for 20 weeks.

	Individual final dry biomass			
	% Sum square	df	F value	*p*
Species	29.6	5	3.821	0.006
Temperature	0.002	1	0.001	0.97
Species :Temperature	3.6	5	0.466	0.80
Residuals	66.7	43		

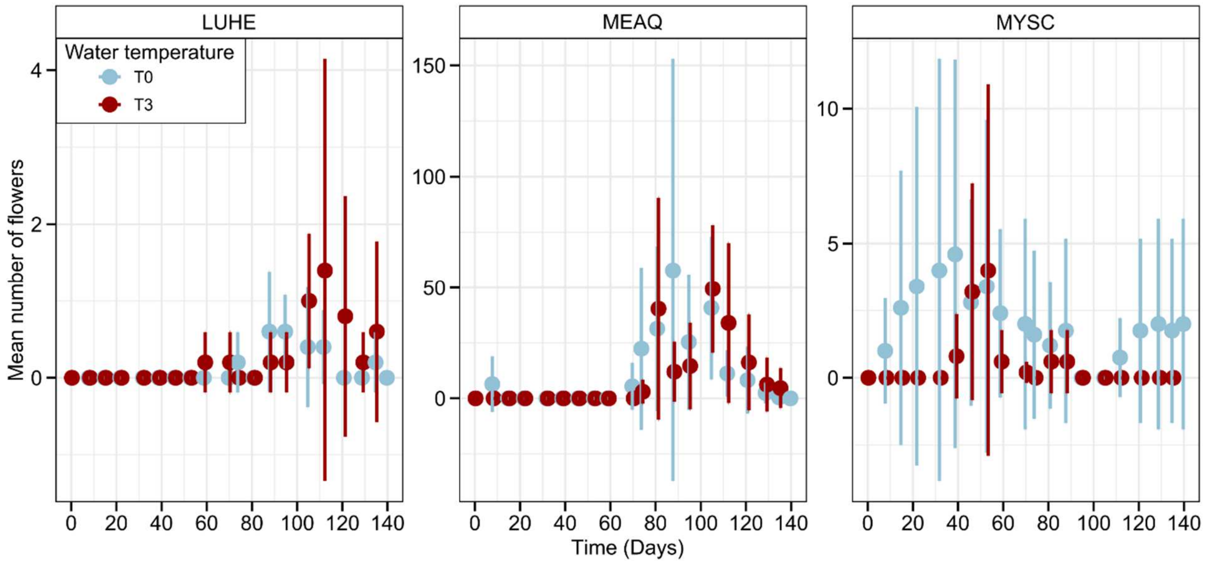

Figure S1. Mean number of flowers (± 95% CI) for three emergent species exposed to two temperature treatments over 20 weeks. n=5.

Plants

Table S2. ANOVA results for chlorphyll, antocyanins and flavonols contents and on Nitrogen Balance Index (NBI) in three species exposed to two temperature treatments for 20 weeks.

		% Sum square	df	F value	*p*
Chlorophyll	Species	79.1	2	40.45	<0.001
	Temperature	0.1	1	0.13	0.72
	Species :Temperature	1.2	2	0.59	0.56
	Residuals	19.6	20		
Anthocyanins	Species	52.8	2	19.76	<0.001
	Temperature	10.1	1	7.59	0.012
	Species :Temperature	10.4	2	3.89	0.037
	Residuals	26.7	20		
Flavonols	Species	81.1	2	82.85	<0.001
	Temperature	6.6	1	13.42	0.001
	Species :Temperature	2.6	2	2.65	0.095
	Residuals	9.8	20		
NBI	Species	32.5	2	6.03	0.009
	Temperature	11.2	1	4.14	0.055
	Species :Temperature	2.6	2	0.47	0.63
	Residuals	53.8	20		

Table S3. ANOVA results for total soluble sugars in six species exposed to two temperature treatments for 20 weeks.

	Total soluble sugars			
	% Sum square	df	F value	*p*
Species	88.0	1	50.38	<0.001
Temperature	3.0	5	296.03	<0.001
Species :Temperature	0.2	5	1.05	0.389
Residuals	8.7	147		

Plants

Supplementary Material 3 – Collection sites

Table S4. Location of sites where fragments from six species were collected

Species	Life-form	Invasion status	Name of waterbody, City	GPS coordinates
Egeria densa	Submerged	Invasive	Etang communal, Saint Martin sur Oust	47.746001, -2.251094
Ludwigia hexapetala	Emergent	Invasive	Etangs d'Apigné, Rennes	48.093609, -1.739927
Mentha aquatica	Emergent	Native	Etangs d'Apigné, Rennes	48.095357, -1.738908
Myosotis scorpioides	Emergent	Native	Etangs d'Apigné, Rennes	48.095482, -1.741242
Myriophyllum brasiliense	Emergent	Invasive	Etangs d'Apigné, Rennes	48.093609, -1.739927
Potamogeton crispus	Submerged	Native	Ruisseau du Lind, Le Rheu	48.091239, -1.783154

Supplementary Material 4 – Details about experimental setup

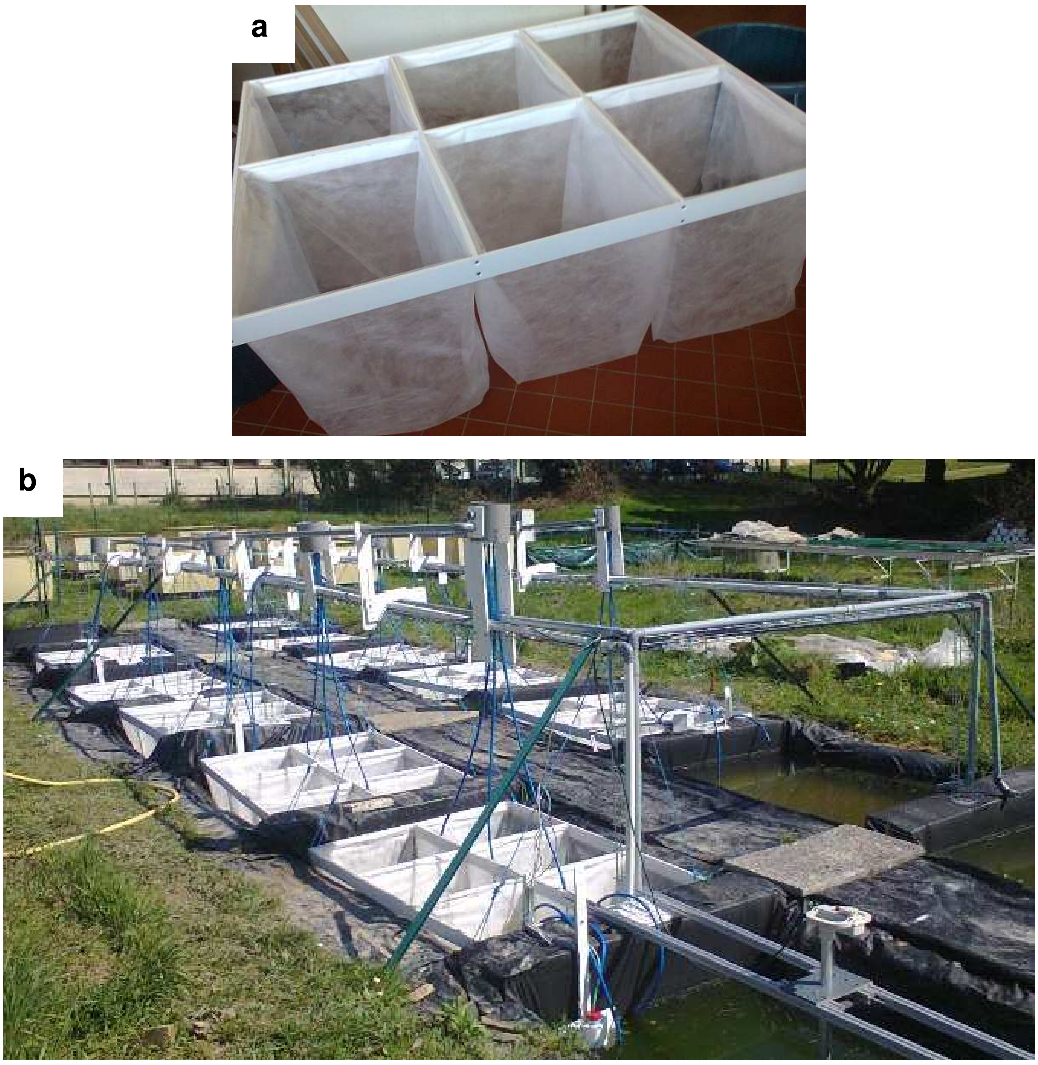

Figure S2. System used to separate mesocosms in six sub-units (a) and overview of the experimental garden (b).

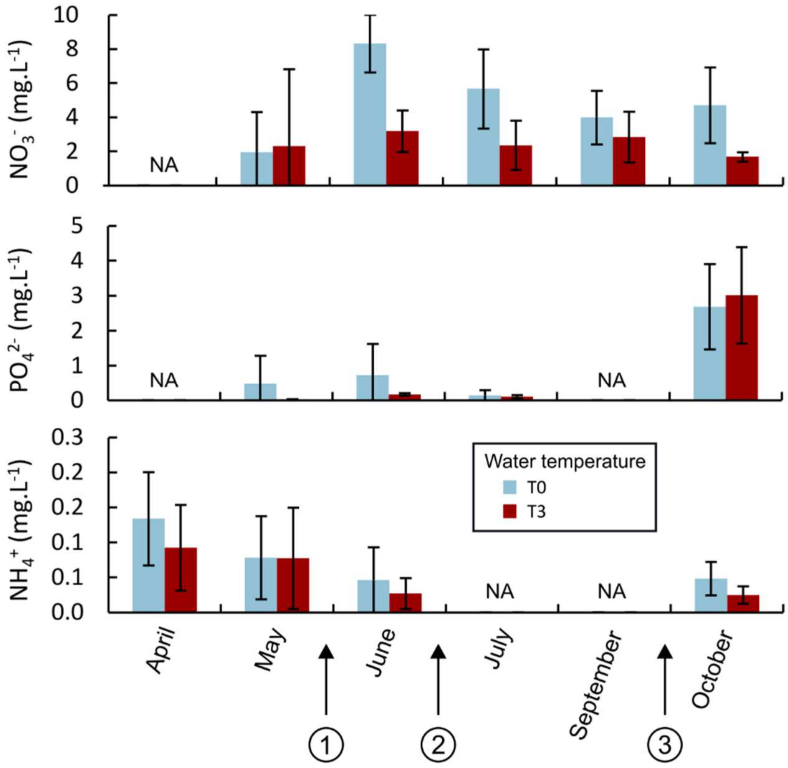

Figure S3. Mean concentration in nitrates, phosphates and ammonium (± confidence interval 95%) of water in mesocosms at two temperature treatments (T0 = ambient temperature, T3 = ambient temperature +3°C), from plant installation to harvest. Circled numbers indicate the addition of liquid fertilizer. n=5.
